# Hospital Festivals, Meetings, &c.

**Published:** 1894-03-24

**Authors:** 


### HOSPITAL FESTIVALS, MEETINGS, ETC.

### THE HOME HOSPITALS ASSOCIATION.

The sixteenth annual meeting of the Governors and members of the above Association was held at Fitzroy House, on March 15th. The annual report stated that during the past year 283 patients and 55 friends had been admitted, the average length of stay of each patient being 17 days. They all expressed themselves satisfied with the treatment received during their stay, and from not one was a complaint received. The Association is in a satisfactory condition financially, the total receipts for the year having amounted to £5,340, and the total expenses to £5,241. The Chairman (Mr. Christopher Heath, F.R.C.S.), in speaking of the advantages of the institution, referred to the time when patients requiring an operation had no other course open but to take lodgings in London; and though many nursing homes at present existed, he was inclined to think that Fitzroy House would compare favourably with most of them, since it had been established with no view to commercial advantage, and was therefore conducted on very liberal lines. He then spoke of the services rendered by the Lady Superintendent (Miss Pearson), and of the excellence of the nursing. He was always sure, he said, that if a nurse was sent from this hospital to a private case she would be trustworthy and capable. He was glad to be able to state that in future the maximum salary to be obtained by the nurses of the institution would be £41 instead of £35 as formerly. Among the other speakers were Mr. Almond-Hind, Mr. H. F. Cox, Dr. T. Robinson, and Sir Richard Quain, who proposed the thanks of the meeting to the medical staff. There was a fairly large attendance at the meeting, most of those present being ladies.

### ROYAL HOSPITAL FOR DISEASES OF THE CHEST.

The eightieth annual court of governors of the Royal Hospital for Diseases of the Chest, City Road, was held on March 14th. The chair was taken by the Lord Mayor, and among those present were Lord Charles Bruce, Mr. T. A. De la Rue, Mr. Hope Morley, Colonel Makins, Dr. Gilbart Smith, Rev. C. Devereux, and Colonel Birch. The report presented to the governors showed a slight decrease in the number of patients treated during the past year, due to the hospital having been closed for repairs during August and September. The "Albert Edward" and "Shaftesbury" wards, which had been closed for want of funds, were reopened on December 2nd, thus raising the number of available beds from fifty to eighty. This has entailed additional expenses on the institution, to meet which increased subscriptions are needed. The expenditure for 1893 amounted to £7,321 1s. 7d., being exceptionally heavy on account of the extraordinary repairs required; the income to £7,862 3s. 8d. The adoption of the report was moved by the Lord Mayor and seconded by Dr. Gilbart Smith, who alluded to the excellent management of the hospital. Among the other speakers were Mr. De la Rue, who proposed a vote of thanks to the honorary medical officers; the Rev. C. Devereux and the Rev. Philip Gast, who both bore witness to the good work done by the institution among the very poor; and Lord Charles Bruce, who expressed the thanks of the court to the Lord Mayor for acting as chairman.

### ST. MARY'S HOSPITAL.

The annual meeting was held at this hospital on March 16th, Colonel S. G. Bird being in the chair. The report for the past year chronicled a great increase in the work of the hospital, the number of in-patients having reached 4,056, while on several occasions there were in the hospital more than the 281 which the wards are arranged to contain. Altogether 39,648 patients were treated during the year, a number greater by nearly 5,000 than in 1892. On the other hand the income of the charity has seriously decreased, the falling off being principally in the item of legacies, which only amounted to £3,047, as against more than £10,000 in the preceding year, and in consequence the receipts of the hospital last year were smaller than at any time since 1885. The Chairman, in moving the adoption of the report, spoke of the very unsatisfactory financial condition of the hospital. The revenue of the institution had not grown, he said, with its increasing work. For instance, the district of Kensington, which sent very many patients to the hospital, contributed very little to its support. He urged that the clergy of all denominations should be invited to co-operate in helping them out of their present difficulties. He believed that, in spite of the apparent expensiveness of the management, there was no other hospital where the patients were expected to provide nothing for themselves, which could receive so many patients per bed, an average of 16 per annum, at so cheap a rate as was done at St. Mary's. The Rev. C. J. Ridgway, in seconding the chairman's motion, said that although he was not prepared to see voluntary hospitals entirely taken over by the State, yet he could not help thinking that they should receive Government grants. He was of opinion that it was not altogether an advantage to their hospital that it was situated in a well-to-do district, since people took it too much for granted that subscriptions and donations would be readily obtained. The thanks of the meeting were then given to the honorary medical staff and to the chairman. Among those present were Mr. A. J. Pepper, F.R.C.S., Dr. Handfield Jones, Dr. Scanes Spicer, Dr. Hubbard, Mr. Parker Young, Mr. J. R. Mellor, Colonel Blair, and Mr. Edward Smith.

### CHELSEA HOSPITAL FOR WOMEN.

A special meeting was held on March 19th at the above hospital, in order to consider the course to be adopted with regard to the imputations cast on the hospital and its medical officers by Dr. Louis Parkes, in his report to the Chelsea Vestry. This report was read to the meeting, as well as the letter sent in reply to the Vestry by the Board of Management, which characterized Dr. Parkes' report as "sensational and inaccurate." The Chairman (Earl Cadogan) then said that he thought it would be wiser at present for the governors to refrain from expressing any opinion on the merits of the case. An inquiry was an absolute necessity, and as the applications of the Vestry to the Home Office and the Local Government Board had not been responded to, he thought they should themselves invite the investigation of a committee of gentlemen in whom the public would have every confidence. He suggested that such a committee might be formed by asking the College of Surgeons, the College of Physicians, and the Chelsea Vestry to nominate each a member; Sir Spencer Wells was absent from England, but Mr. Jonathan Hutchinson, their other consulting Surgeon, and Dr. Robert Barnes, their consulting Physician, should, he thought, be asked to join; and he further suggested the names of two "laymen"—Lord Sandhurst and Sir Charles Dilke, as likely to be of great service to such a committee. Sir Algernon Borthwick having moved that the Chairman's suggestion be adopted, the Rev. Webb Peploe proposed that Lord Balfour of Burleigh's name should be substituted for Sir

Charles Dilke's; when Dr. Fancourt Barnes proposed that both names should be allowed to stand, which was ultimately agreed to. Mr. Hutchinson said he should approve of the course proposed by the Chairman if he thought the present crisis grave enough to require such a measure. There were three charges against the hospital. The scarlet fever charge he dismissed as a mere nothing, while as for the drains, they had been altered in accordance with the recommendations of Dr. Parkes (which were, however, at variance with the plans of many sanitary authorities). The most serious charge was that which concerned the alleged excessive mortality. In reply to that, he would say that not only was last year an unfortunate one, but that many of the deaths were those of patients who had been brought to the hospital in a very advanced stage of illness, and, but for the sake of humanity, would not have been admitted at all. He held that Dr. Parkes' charges were sufficiently refuted by the reply sent to the Vestry and the letters published in the medical journals. He was seconded by Dr. Robert Barnes, who said that he had no objection to an inquiry, but saw no need of it. On the other hand, Dr. Inglis Parsons, who was seconded by Dr. Fenton, spoke strongly in favour of an inquiry, which would vindicate the medical staff from the charges brought against them. The Chairman said that, although he was glad that Mr. Hutchinson had such confidence in the institution as to think an inquiry unnecessary, he was of opinion that investigation must take place as soon as possible, in which opinion the meeting unanimously concurred.

### THE DENTAL HOSPITAL OF LONDON.

The thirty-sixth annual meeting of this institution was held at the hospital in Leicester Square, on Thursday, March 15th, under the presidency of J. Smith Turner, Esq., Vice-President. The annual report having been unanimously adopted, the Chairman, in an able address, called attention to the satisfactory report for the year 1893. He urged very strongly the claims of the proposed new hospital, pointing out that from the insanitary and overcrowded state of the building, a new hospital was much needed; he further called attention to the great value of the institution as a teaching centre, from which excellently equipped dental surgeons are turned out, so that the institution was valuable, both as a charity and an educational establishment, and deserved therefore the hearty support of the public. Towards the £40,000 required for the new building, £9,700 has been promised or collected; of this sum £2,000 only has been contributed by the public, the remainder having been subscribed by the dental profession. The Chairman ventured to think this should not be, and that the public should contribute more liberally towards so useful and deserving an object. For so deserving a charity, the support so far given is certainly very small, and it is to be hoped that the charitable public will generously respond to the appeal. Help by an increase in annual subscriptions is also earnestly solicited. The report gave the number of operations of all kinds performed in the hospital during the past year as 55,325. It was also stated that there was a slight increase in the annual subscriptions (£1,053 0s. 6d., as against £1,013 4s. 6d. in 1892), though the total amount received for the General Fund had somewhat fallen off, owing to the donations specially given to the Building Fund.

### ROYAL SEA BATHING INFIRMARY.

The general court of governors of the Royal Sea Bathing Infirmary for Scrofula was held on Monday, March 12th, at 30, Charing Cross. There were present: The Rev. Richard Whittington, Mr. T. Dunn, Mr. P. S. Euston, Mr. Michael Biddulph, M.P., J.P., and others. The report presented to the court chronicled an excess of income over expenditure of £247 4s. 5d., due, however, solely to the legacies received during the year. The dependable income of the charity continues to be below the expenditure, and the directors see no prospect at present of opening more than the 8J beds at present in use—140 being closed for want of funds. In all 445 patients were treated during the past year, of whom 83 were in the hospital at the beginning of the year, and 362 have been since admitted. The percentage of cured has been 28·9, per cent.; of those greatly benefited 44·2 per cent., while there has been but one death. Allusion was made by Mr. Whittington to the death of the late treasurer, Mr. Ruthven Pym; and Mr. M. Biddulph was unanimously elected in his stead.

### THE ROYAL HOSPITAL FOR CHILDREN AND WOMEN.

The annual meeting of the Governors and friends of this hospital was held at the Mansion House on March 16th, the Lord Mayor presiding. The annual report gave the number of in-patients during the year as 593, and of out-patients as 7,488, an increase in both cases over the numbers of the preceding year. The subscriptions have somewhat fallen off, but the donations on the other hand have increased, and the liabilities of the hospital have been reduced from £800 in 1892 to £500 in December, 1893, this reduction being, to a great extent, due to a gift of £700 from Baron de Hirsch. The Lord Mayor, in addressing the meeting, said that the efficiency and good management of the hospital were fully vouched for by the high place which it held in the list of the Hospital Saturday and Sunday Funds. It was decided to open a subscription list for the especial purpose of meeting the deficit that still existed.

### HOSPITAL AND HOME FOR INCURABLE CHILDREN.

The annual meeting of this hospital was held at 2, Maida Vale, on March 17th. The report announced a serious falling off in the income of the institution, the total receipts having only amounted to £845 5s. 3d. as against £1,112 for 1892, while it was found impossible to bring the expenses below £1,186 2s. 2d. The total number of patients under treatment during the year has been 36; there has been one death, but on the whole the general health of the children, with the exception of a few cases of chicken-pox, has been good. The adoption of the report was moved by the Rev. Canon Duckworth, the chairman. He spoke with regret of the unsatisfactory financial condition of the hospita . He was seconded by Lieut.-General Lowry, C.B., who urged that strenuous efforts should be made in support of the institution by its friends, and bore testimony to the comfort and happiness of the children at the home. A vote of thanks to Mr. F. E. Webb, hon. medical officer, and to Miss Coleman, the matron, was passed; and Mr. Hoblyn, in returning the thanks of the meeting to the chairman, hoped that Canon Duckworth would see his way to giving them another offertory this year, to which the Chairman replied that in spite of the many applications for offertories he received he would do his best to procure one. There were present: Mr. F. Aylmer Lloyd (hon. treasurer), Mr. W. H. Ash, Mr. S. Fisher (hon. secretary), and several ladies.

### MARYLEBONE GENERAL DISPENSARY.

The re-opening of this dispensary by the Duke and Duchess of Fife took place on March 16th. The Duke and Duchess were received by the Bishop of Marlborough, Canon Duckworth, Canon Barker, Sir Horace Farquhar, Sir F. Seager Hunt, M.P., and Mr. Christopher Eales, and escorted to the waiting hall, where an address was read by Dr. C. J. Hare describing the chief features of the institution, particularly the provident system and the visiting of the sick in their own homes. He stated that £2,000 are still required to clear off the debt on the new building. The Duke of Fife, in replying to the address, referred to the long time—over a hundred years—that the institution had been carrying on its merciful work among the poor. He then declared the buildings open, in the name of the Duchess, who, he said, did not belong to the talking sisterhood, and had requested him to perform the ceremony for her. Before leaving the Duke and Duchess inspected the building, during which time a selection of music was performed to the visitors in the waiting hall.

